# Preparation and Characterization of Fe_3_O_4_ Particles with Novel Nanosheets Morphology and Magnetochromatic Property by a Modified Solvothermal Method

**DOI:** 10.1038/srep09320

**Published:** 2015-03-23

**Authors:** Lin Zhuang, Wei Zhang, Yongxin Zhao, Hui Shen, Han Lin, Jinhua Liang

**Affiliations:** 1 State Key Laboratory of Optoelectronic Materials and Technologies, Guangdong Provincial Key Laboratory of Photovoltaics Technologies, School of Physics and Engineering, Sun Yat-Sen University, Guangzhou 510275, P. R. China; 2 School of Physics and Optoelectronic Engineering, Guangdong University of Technology, Guangzhou 510006, China; 3 School of Chemistry and Chemical Engineering, Sun Yat-Sen University, Guangzhou 510275, P. R. China; 4 Department of Orthodontics, Guanghua School of Stomatology, Hospital of Stomatology, Sun Yat-sen University, Guangzhou 510055, P.R. China

## Abstract

Novel-morphological Fe_3_O_4_ nanosheets with magnetochromatic property have been prepared by a modified solvothermal method. Such nanosheets could form one-dimension photonic crystal under an external magnetic field. The Fe_3_O_4_ nanosheets suspension could strongly diffract visible light and display varied colors with changing the intensity of the magnetic field. The photonic response is rapid, fully reversible and widely tunable in the entire visible spectrum. Excellent magnetic properties of these Fe_3_O_4_ nanosheets are exhibited with a high saturation magnetization (82.1 emu/g), low remanence (13.85 emu/g) and low coercive force (75.95 Oe). The amount of the solvent diethylene glycol (DEG) plays a key role in the formation of the sheet-shaped morphology. When the ratio of the DEG reaches 100%, the growing of the crystal plane (111) of Fe_3_O_4_ is inhibited and the sheet-like Fe_3_O_4_ crystals are formed.

Nanostructure magnetic Fe_3_O_4_ particles are of much interest because of their applications in a variety of areas such as ferrofluid, advanced magnetic materials, magnetic recording media and biomedical purpose, etc^1^,^2^,^3^,^4^,^5^,^6^,^7^. With the advancement of magnetic materials, especially in the area of the magnetic responsive photonic crystal, considerable efforts have been attempted to improve their magnetic and optical properties. Different from other external stimuli systems of responsive photonic crystal, introducing magnetic components into the photonic crystal makes it more convenient and precise to control the properties through an external magnetic field. Such magnetic responsive photonic crystals have been demonstrated in the applications of different color display units including dynamic displays, rewritable photonic papers and full color printing systems.

Recently, a high-temperature hydrolysis method was reported to synthesize monodisperse superparamagnetic Fe_3_O_4_ particles with the nanosphere morphology^7^. These Fe_3_O_4_ nanospheres could form one-dimension photonic crystal in aqueous and non-polar media, which could diffract the light of visible wavelengths. As Known,the magnetic properties of nanoparticles are greatly dependent on their morphologies. Varied morphologies of Fe_3_O_4_ nanocrystals including nanotubes^8^, hollow spheres^9^, nanochains^10^, nanoflowers^11^ and nanosheets^12^ have been fabricated. Compared to magnetite particles with other shapes, nanosheets have better crystallinity and smaller lattice distortion, which enable them possess better physical and magnetic properties. To the best of our knowledge, the synthesis of Fe_3_O_4_ nanosheets with the magnetochromatic property has hardly been reported.

Numerous chemical methods including microemulsions^13^, sol-gel syntheses^14^, sonochemical reactions^15^, hydrothermal reactions^16^, hydrolysis and thermolysis of precursors^17^ have been used to synthesize Fe_3_O_4_ magnetic nanoparticles. In the specific synthesis of nanosheets, the methods of solvothermal process^12^, solid-state thermal decomposition route^18^, supercritical fluid technique^19^ and bottom-up technique^20^ have been attempted. These synthesis processes are usually involved in one-step reaction, and the surface modification procedure is incorporated with the synthesis of particles. In addition, in the conventional synthesis methods, deoxygenated protection and precisely control of the pH value are required.

Here we report a two-step solvothermal method to synthesize the Fe_3_O_4_ nanosheets with good magnetochromatic property without deoxygenated protection and controlling of the pH value. In the process, the surface modification of the Fe_3_O_4_ particles is performed after synthesizing the particles. The separation of these two steps simplifies our synthesis method and increases the reproducibility of the obtained nanosheets. In addition, there are more choices for capping agents since the modification step is independent. When an external magnetic field is introduced to the Fe_3_O_4_ nanosheets suspension, the particles suspension could diffract light with different wavelength and display various colors under magnetic fields with different intensity. The photonic response of the magnetic Fe_3_O_4_ nanosheets is reversible and widely tunable in the visible spectrum, indicating great potential applications in sensors and optical devices.

## Results and Discussion

**Figure 1:**
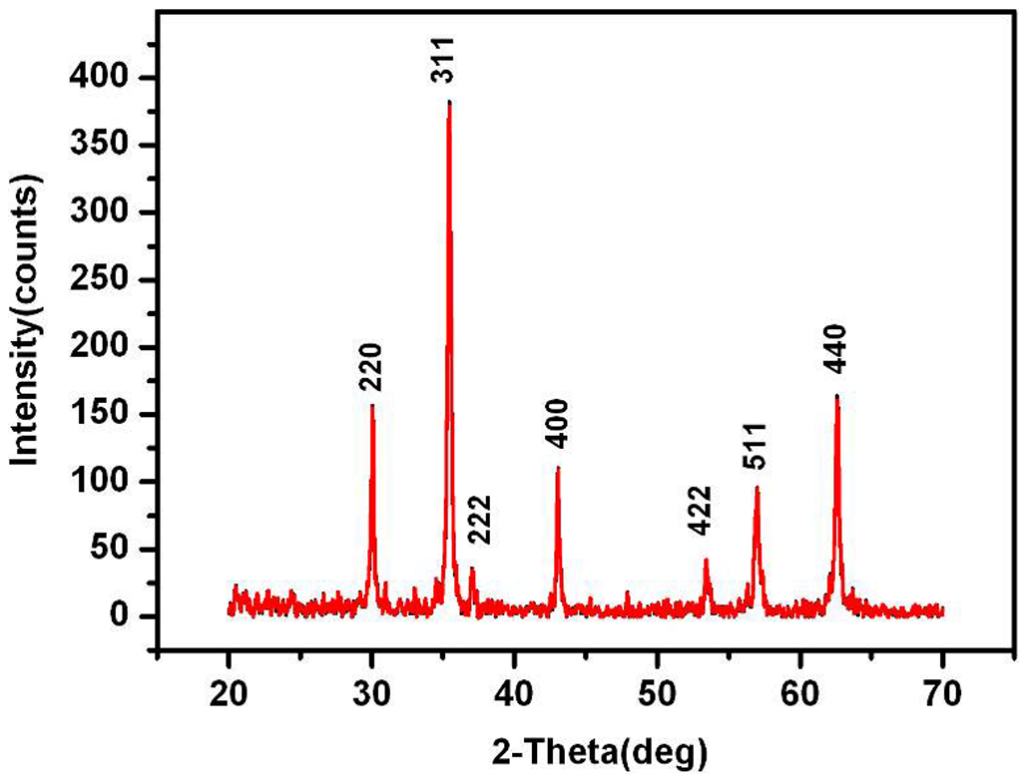
XRD pattern of the Fe_3_O_4_ nanosheets.

The X-ray diffraction pattern of the Fe_3_O_4_ nanosheets is shown in Fig. 1, which reveals the formation of magnetite with well-defined crystallinity via the modified solvothermal process. All of the diffraction peaks match well with the normal characteristic diffractions of the Fe_3_O_4_ inverse spinel structure (PCPDFWIN v.2.02, PDF No. 89-0691). The calculated cell parameter (8.3915 Å) is comparable with the reported value. No obvious XRD peaks arising from impurities are found, which indicates no evidence that Fe_3_O_4_ particles are contaminated by foreign materials in the system.

**Figure 2:**
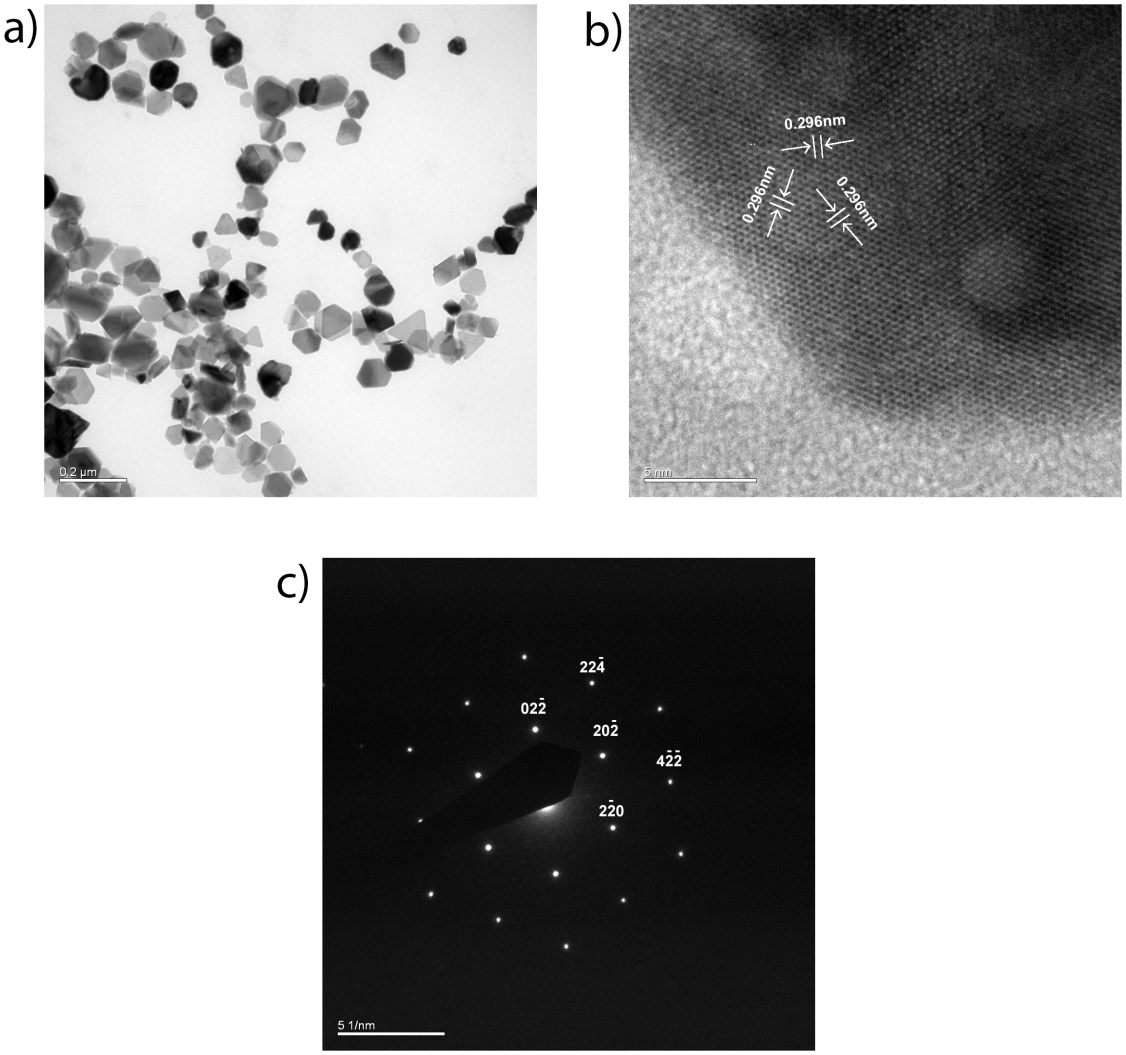
(a) Low magnitude TEM image of Fe_3_O_4_ nanosheets; (b) HRTEM of Fe_3_O_4_ nanosheets; (c) SAED pattern of Fe_3_O_4_ nanosheets. The scale bars are 0.2 μm, 5 nm, and 5 1/nm respectively.

In Fig. 2(a), the Fe_3_O_4_ nanosheets are of regular hexagonal or triangle structure with the edge length of 80–150 nm. The detailed crystalline information of the particles is shown in Fig. 2(b) and Fig. 2(c). The HRTEM image of selected area in the sample in Fig. 2(b) indicates that the nanosheets have well-defined crystallinity with an interplanar spacing of 0.296 nm. The diffraction points array tidily along a certain direction in the selected area electron diffraction (SAED) pattern as shown in Fig. 2(c). Crystal planes of (220) and (422) could be found in Fig. 2(c), which are consistent with the XRD pattern in Fig. 1. From Fig. 2(b) and Fig. 2(c), it's clear that the growth rate of the crystal plane (111) is depressed, which is the key factor for the formation of the hexagonal or triangle shape of the nanosheets.

**Figure 3:**
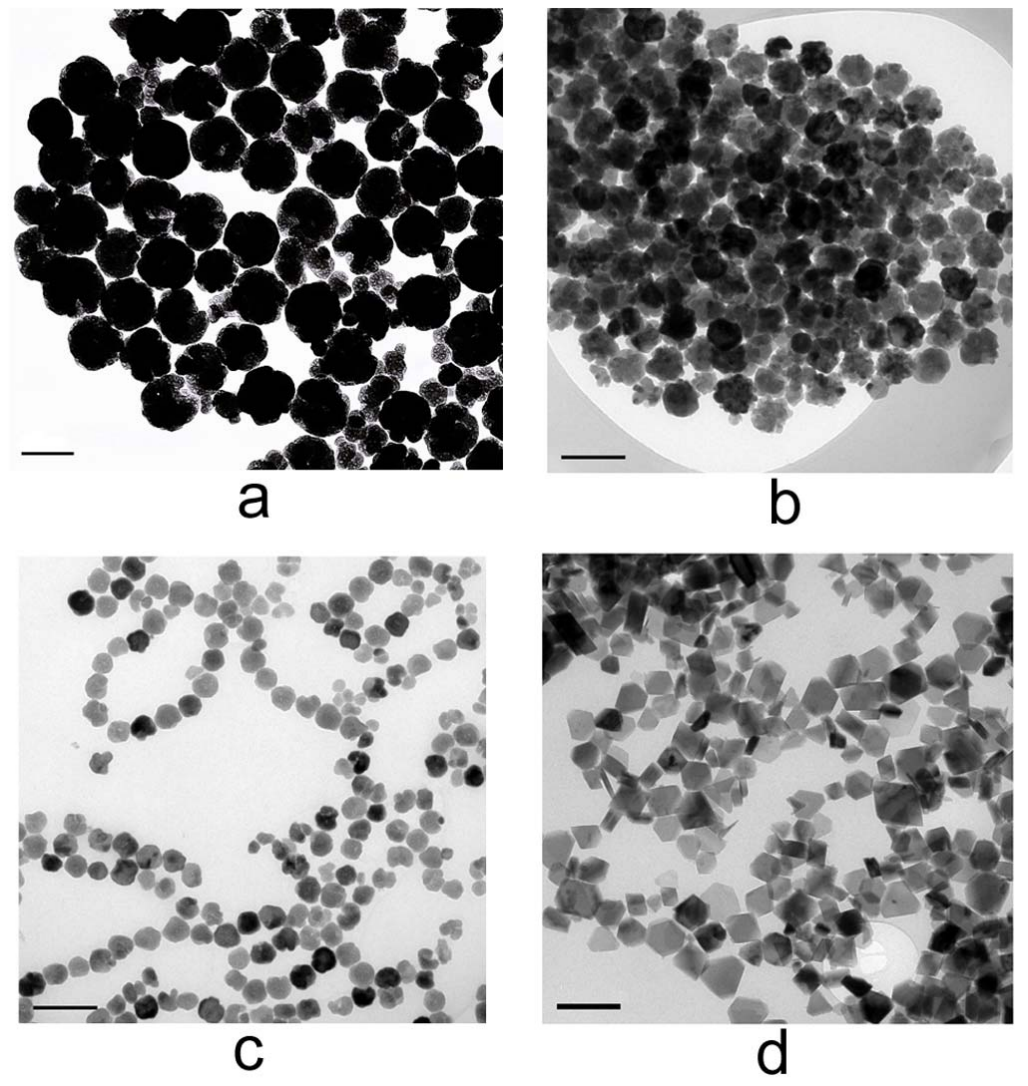
Low magnitude TEM images of Fe_3_O_4_ particles: (a) 180 nm particles (DEG/EG = 0/40); (b) 100 nm particles (DEG/EG = 26/14); (c) 60 nm particles (DEG/EG = 30/10); (d) nanosheets (DEG/EG = 40/0). All the scale bars are 200 nm.

The nucleation of the Fe_3_O_4_ particles could be well controlled by changing the volume ratios of the solvent DEG and Ethylene Glycol (EG). The amount of the DEG plays a key role in the formation of the sheet-shaped Fe_3_O_4_ nanostructures, which can be confirmed by the comparison experiments with different concentrations of DEG. Fig. 3 shows that when the volume ratios of DEG/EG (v/v in mL) are 0/40, 26/14, 30/10 and 40/0, different sizes and shapes of Fe_3_O_4_ particles have been obtained. The diameters of the nanospheres are 180 nm (Fig. 3a), 100 nm (Fig. 3b) and 60 nm (Fig. 3c), and the shape of the nanosheet is shown in Fig. 3d. The particle size decreases as the ratio of DEG/EG increases. This size tunability may due to the different diffusion coefficient of the ions in the solution caused by the varying concentrations of the DEG. Since DEG has a stronger affinity to the Fe^3+^ ions, the viscosity of the solvent increases as the portion of the DEG increases and therefore the diffusion coefficient of the ions in the reaction system decreases, which leads to smaller grain size of the Fe_3_O_4_ particles and inhibits the formation of the secondary structure. In the solvothermal process, when the ratio of the DEG is rather low, the formation of the microstructure features after fast nucleation in solution relates to the aggregation growth process and the Ostwald ripening process. When the ratio of the DEG reaches 100%, the mechanism of the crystal growth is different from the former and the morphology of the final product becomes nanosheet.

**Figure 4:**
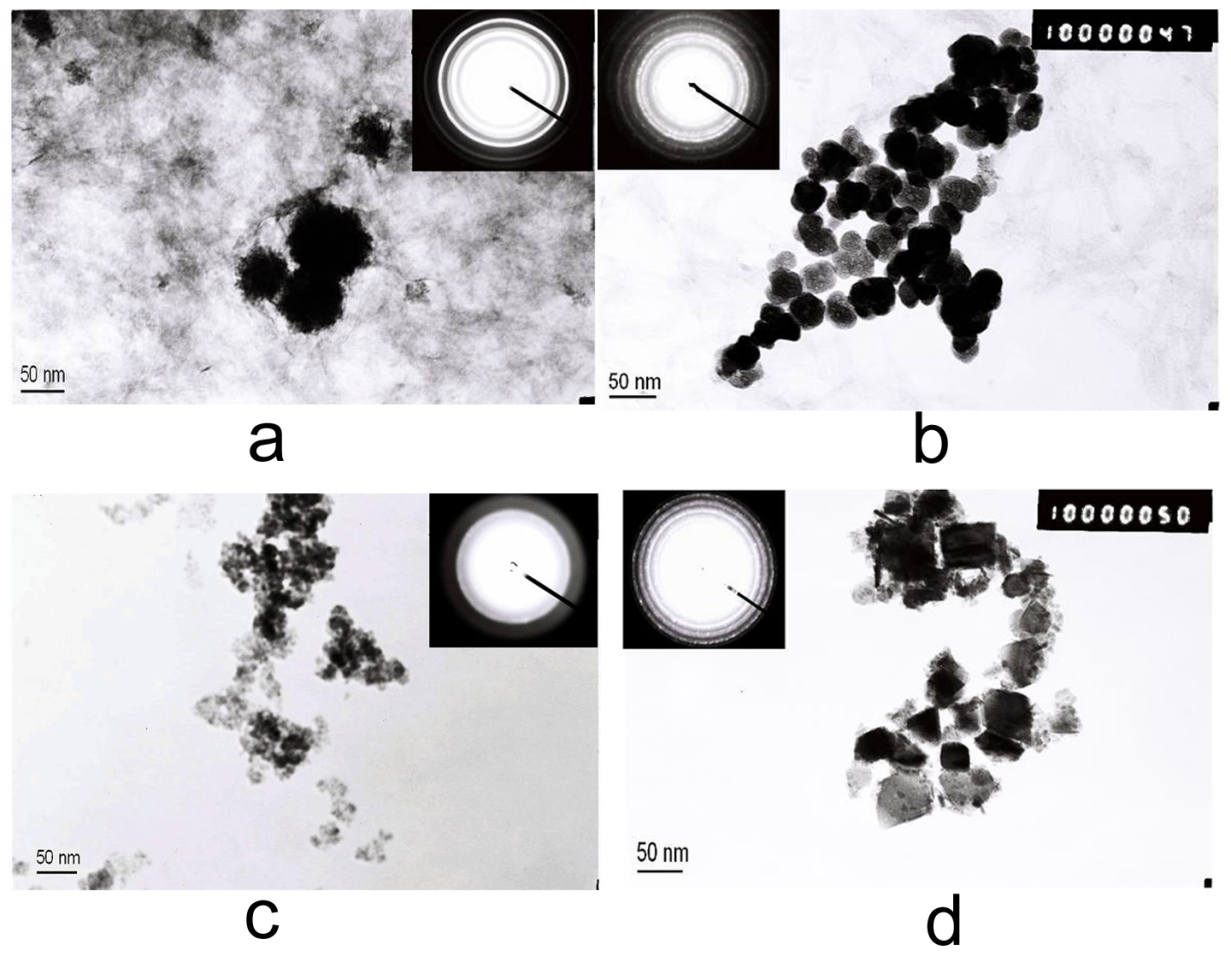
Low magnitude TEM images of Fe_3_O_4_ product under the conditions of different reaction time and percentage of DEG in the solvent: (a) 1 hour, DEG% = 65%; (b) 3 hours, DEG% = 65%; (c) 1 hour, DEG% = 100%; (d) 3 hours, DEG% = 100%.

To further understand the formation mechanism of the Fe_3_O_4_ nanosheets, a number of matrix precursor experiments were carried out under different reaction time and different concentrations of DEG. TEM images in Fig. 4(a) and Fig. 4(c) reveal that the samples consist of unconsolidated particles when the reaction time is 1 h. These unconsolidated particles only congregate but not form the crystal structure according to the electron diffraction patterns in Fig. 4(a) and Fig. 4(c). Fig. 4(b) reveals that the nanospheres preliminarily form after reaction for 3 h. In the reaction system, the Fe (III) compounds hydrolyze and deoxidize to Fe(II) compounds partially. Fe_3_O_4_ crystal nucleus are formed by the interaction of Fe(II) and Fe(III) hydrates. The growing of the crystal follows the process of homogeneous nucleation and the final product of Fe_3_O_4_ nanospheres is obtained. Fig. 4(d) shows the formation of the nanosheets. When the ratio of the DEG reaches 100%, DEG and Fe^3+^ ions complex and form a steady solution. The growing of the crystal and aggregation of nanoparticles are slow under the strong complexation and the high viscosity of the solvent. The growth of the crystal plane (111) is limited and the shape of the nanosheets, but not the nanospheres, is formed.

**Figure 5:**
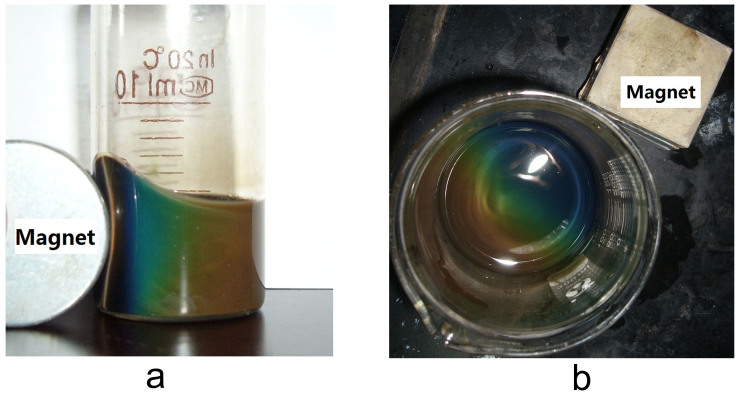
Photographs of Fe_3_O_4_ nanosheets colloidal solution formed in response to an external magnetic field. (a) and (b), the external magnetic field are both set beside the Fe_3_O_4_ nanosheets suspension.

**Figure 6:**
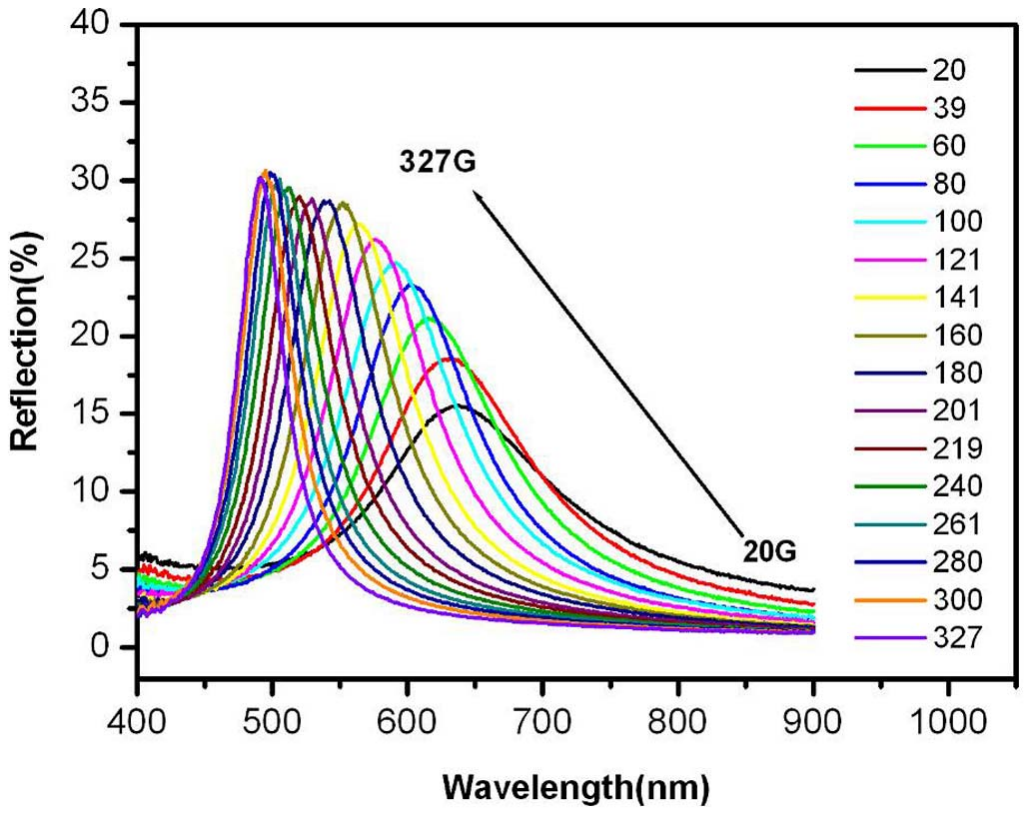
Reflection spectra of one-dimension photonic crystals formed by Fe_3_O_4_ nanosheets in aqueous solution in response to external magnetic field with different intensity.

The Fe_3_O_4_ sheet-like nanoparticles exhibit good magnetochromatic properties. The photographs and reflection spectra of the aqueous solution of Fe_3_O_4_ colloidal particles (ca. 15.00 mg mL^−1^) in response to an external magnetic field are shown in Fig. 5 and Fig. 6. When a magnetic field is applied to the nanosheets suspension, the magnetic colloid displays clearly a gradient of colors. The particles in the suspension form a chain-like structure along with the direction of the external magnetic field, which is known as photonic crystal^21^. The structure formed by the magnetic nanosheets could diffract visible light of different wavelength ranging from 480 to 640 nm as shown in Fig. 6. The reflection rate increases and the diffraction peaks blue shift as the magnetic field is increased from 20 to 327 G. The low reflection rate and broad peak width at a low magnetic field are due to the Brownian motion, which significantly interferes with the formation of the long-range order structure. When the magnetic field increases, the magnetic interaction between two magnetic nanoparticles surpasses the Brownian motion and the chain-like structure is enhanced, which makes the diffraction peak sharper and higher.

**Figure 7:**
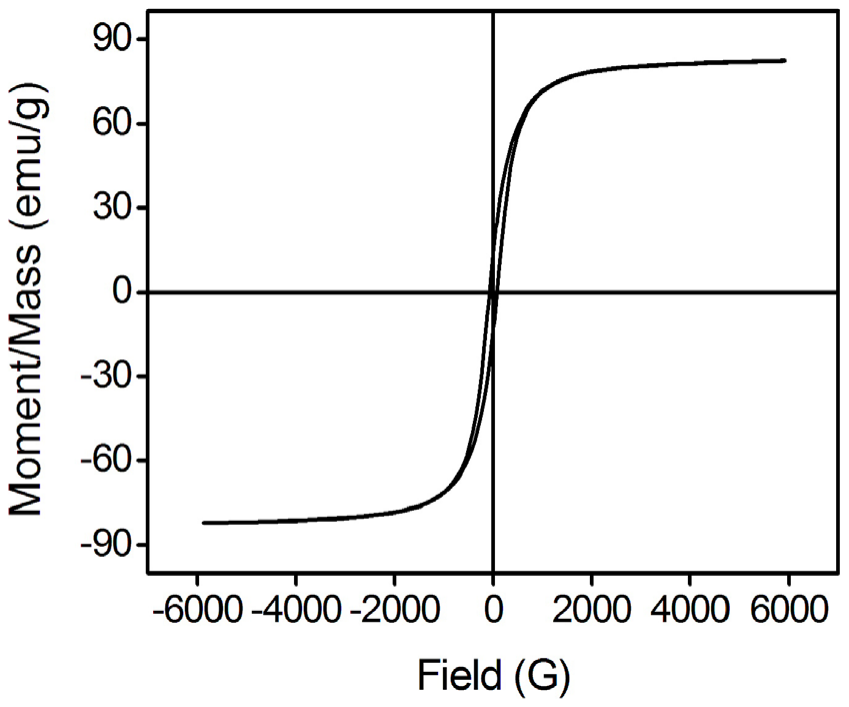
Magnetization curve of Fe_3_O_4_ nanosheets at room temperature.

**Figure 8:**
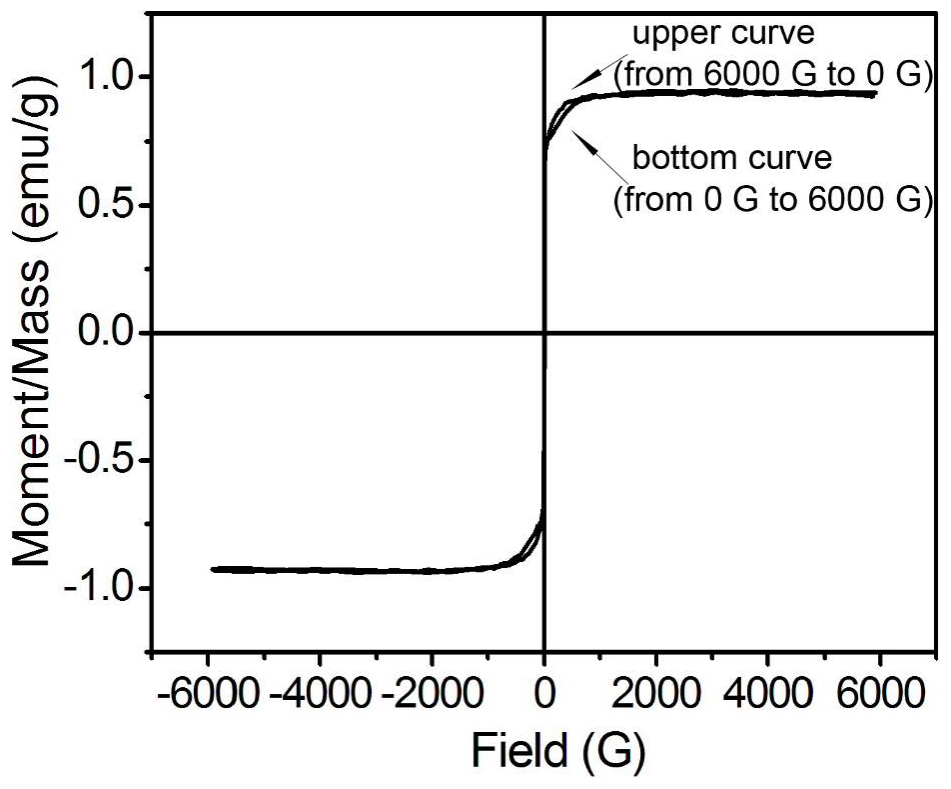
Magnetization curve of Fe_3_O_4_ nanosheets suspension (concentration 15 mg/mL).

The magnetic properties of the modified nanosheets and the corresponding nanosheets suspension were investigated respectively. As shown in Fig. 7, the Fe_3_O_4_ nanosheets exhibit soft magnetism with a low remanence (13.85 emu/g) and low coercive force (75.95 Oe). The saturation magnetization of the magnetic nanosheets is 82.10 emu/g, which is much higher than that of the nanoparticles prepared by the common chemical coprecipitation method (about 60–65 emu/g). The high reaction temperature in our modified solvothermal method results in a higher crystallinity and consequently leads to a higher saturation magnetization. While in the observation of the hysteresis loop of the particles suspension as shown in Fig. 8, the colloid exhibits essentially no remanence or coercive force. This interesting result explodes the previous conclusion that ferromagnetic particles cannot be dispersed in aqueous media. The remanence and the coercive force of the Fe_3_O_4_ nanosheets dispersed in aqueous solution are found to decrease to 0.042 emu/g and 1.94 Oe, respectively. This phenomenon indicates that if suitable surface modification is performed to decrease the magnetic force between the adjacent nanosheets, the ferromagnetic particles can rely on the Brownian motion to disrupt the chain-like structure induced by the magnetic moment, and then a transformation from ferromagnetism of the particles to a hysteresis loop with much small remanence and coercive force of the particles suspension appears. In this case, there will be no significant magnetic interactions remaining after removing the external magnetic field, which is important for achieving a reproducible and reversible optical response. The hysteresis loop of Fig. 8 displays irreversibility when the applied external magnetic field is between 100 and 900 G. when the external field is decreased from 900 G in the upper curve, the particles could still keep the saturation magnetization until the field is decreased to be about 700 G. And during the magnetic field is between 900 and 100 G, the magnetization value in the upper curve is clearly larger than that in the bottom curve. This is due to the complex effect of metastable state of the magnetic dynamic nanoparticles and the possible relaxation characteristic of the formed chain-like structure when decreasing the external magnetic field, the Brownian dynamics of particles, and the electrostatic interactions due to the PAA.

In summary, magnetic Fe_3_O_4_ nanosheets have been synthesized by a two-step solvothermal method. The Fe_3_O_4_ nanosheets suspension exhibits magnetochromatic property under an external magnetic field. The formed one-dimension photonic crystals by the Fe_3_O_4_ nanosheets in aqueous solution under external magnetic field could strongly diffract visible light with different wavelength and display various colors. Diffraction peaks blue shift as the magnetic field increases which could cover the entire visible spectrum. The surface modification increases the dispersibility of the nanosheets and achieves a transformation of ferromagnetism of the particles to a hysteresis loop with much small remanence and coercive force of the particles suspension, which enables the optical response of the colloid solution reproducible and reversible. Excellent magnetic properties of the nanosheets have been obtained with a high saturation magnetization (82.1 emu/g), low remanence (13.85 emu/g) and low coercive force (75.95 Oe). The amount of the solvent DEG plays a key role in the formation of the sheet-shaped Fe_3_O_4_ nanostructures. The size of the Fe_3_O_4_ nanoparticles decreases as the ratio of DEG/EG increases in virtue of the strong affinity of DEG to Fe^3+^ ions and the higher viscosity of the solvent. A transformation of the shape from nanosphere to nanosheet appears when the ratio of DEG/EG is 40/0.

## Methods

### Synthesis of polyacrylic acid

125 mL Deionized H_2_O and 1.00 g ammonium persulfate (APS) were added into a three-neck bottle with a dropping funnel and reflux condensing tube. When the APS was dissolved under magnetical stirring, 5.00 g acrylic acid and 8.00 g isopropanol were further added. Then the mixture was heated to 65–70°C, following with the add of a solution containing 40.00 g acrylic acid, 2.50 g ammonium persulfate and 40 mL DI H_2_O drop by drop through the dropping funnel. Due to the heat released in the polymerization reaction, the temperature of the system would rise and lead to reflux. After all the acrylic acid and APS solution reacted for 30 min, the mixture solution was kept refluxing at 94°C for 1 h and then cooled down.

### Synthesis and surface modification of Fe_3_O_4_ particles with sheet-like morphology

Ferrous chloride (FeCl_2_), ferric chloride (FeCl_3_), Sodium Acetate (NaAc) and Polyethylene Glycol (PEG) were dissolved in 40 mL Diethylene glycol (DEG) under stirring and ultrasonic treatment. The molar ratio of FeCl_2_:FeCl_3_:NaAc:PEG was 1:1.9:22:1.25. The homogeneous yellow mixture was then transferred to a Teflon-lined stainless-steel autoclave and sealed to heat at 220°C. It is worth noting that the inert gas protection is not necessary due to the closed system and the reducing environment created by DEG. After reaction at 220°C for 7 h, the autoclave was cooled down to room temperature. The as-prepared Fe_3_O_4_ particles were washed several times with ethanol and DI H_2_O before the surface modification step.

20 mL NaOH solution (1.00 mM) was added into the wet Fe_3_O_4_ precipitation, and then the mixture was ultrasonic treated to disperse the particles into the solution and form the magnetic nanoparticles (MNPs) suspension. 20 mL PAA solution (10.0 g/L) and 1 mL FeCl_3_ (0.1 mol/L) were mixed to form an orange PAA-Fe^3+^ complex solution. The MNPs suspension was slowly added into the PAA-Fe^3+^ complex solution under mechanical stirring. The mixture was ultrasonic treated at 80°C for 15 min, and then the precipitate was collected by magnetic separation. After adding 20 mL NaOH (0.05 mol/L) solution into the precipitate and 10-s ultrasonic treatment of the mixture, the product was collected again by magnetic separation and washed by DI H_2_O three times. Finally, the magnetic colloid solution was obtained by dispersing the product in a certain amount of DI H_2_O. It should be noted that the particles in the suspension are fairly stable in air and could be stored for months.

### Characterization

X-ray diffraction (XRD) patterns were recorded on a Power X-ray Diffractometer (D/Max-IIIA) with Cu Kα radiation. Transmission electron microscopy (TEM) photographs were taken on a high-resolution transmission electron microscope (HRTEM, JEM-2010) at an accelerating voltage of 200 kV. The optical properties of the photonic crystals formed by the magnetic Fe_3_O_4_ nanosheets under external magnetic field, which was controlled by a home-made electromagnet, were collected by using a QE65000-ABS Scientific-grade Optic Fiber Spectrometer (Ocean Optic, Inc.). Room temperature magnetization measurements were performed with a LakeShore Model 7404 vibrating sample magnetometer (VSM).

## Author Contributions

L.Z. wrote the manuscript and analysed the experimental data, W.Z. provided crucial support for the experiments, Y.Z. and J.L. carried out the experiments, analyzed the experimental data, H.S. supervised the project and H.L. helped writing the main manuscript.
